# Regulation of Macrophage and Dendritic Cell Function by Chondroitin Sulfate in Innate to Antigen-Specific Adaptive Immunity

**DOI:** 10.3389/fimmu.2020.00232

**Published:** 2020-03-03

**Authors:** Sonoko Hatano, Hideto Watanabe

**Affiliations:** Institute for Molecular Science of Medicine, Aichi Medical University, Nagakute, Japan

**Keywords:** chondroitin sulfate, glycosaminoglycan, proteoglycan, antigen-presenting cell, receptor type of protein tyrosine phosphatase sigma

## Abstract

Chondroitin sulfate (CS), a type of glycosaminoglycan (GAG), is a linear acidic polysaccharide comprised of repeating disaccharides, modified with sulfate groups at various positions. Except for hyaluronan (HA), GAGs are covalently bound to core proteins, forming proteoglycans (PGs). With highly negative charges, GAGs interact with a variety of physiologically active molecules, including cytokines, chemokines, and growth factors, and control cell behavior during development and in the progression of diseases, including cancer, infections, and inflammation. Heparan sulfate (HS), another type of GAG, and HA are well reported as regulators for leukocyte migration at sites of inflammation. There have been many reports on the regulation of immune cell function by HS and HA; however, regulation of immune cells by CS has not yet been fully understood. This article focuses on the regulatory function of CS in antigen-presenting cells, including macrophages and dendritic cells, and refers to CSPGs, such as versican and biglycan, and the cell surface proteoglycan, syndecan.

## Introduction

Glycosaminoglycans (GAGs) are linear polysaccharides consisting of repeating disaccharide units and modified with sulfate groups at various positions on the sugar residues. The GAG chains retain negatively charged domains due to characteristics of the sulfate groups, allowing for the absorption of water and other positively charged soluble ligands, such as chemokines (1, 2), cytokines (3), growth factors (4, 5), and cell surface receptors (6, 7). They are classified into chondroitin sulfate/dermatan sulfate (CS/DS), heparin/heparan sulfate (HP/HS), hyaluronan (HA), and keratan sulfate (KS).

At the site of injury or infection, macrophages release cytokines to activate endothelial cells, and HS on endothelial cells binds to L-selectins on leukocyte, leading to leukocyte rolling (8). Macrophages also release substantial amounts of chemokines that bind to GAGs at the endothelial surface (9). Leukocytes adhere to endothelial cells firmly and then migrate through the endothelial barrier. Therefore, the roles of GAGs in inflammation and immunity are linked to chemokines due to their highly polar nature. HA is best studied in clinical applications for its influence on inflammation, and its role is varying depending on its molecular weight. High-molecular-weight HA has anti-angiogenic, anti-inflammatory, and immunosuppressive effects (10). Conversely, low-molecular-weight HA promotes angiogenesis, inflammation, and cell migration (10, 11). HA forms provisional matrices with a CS proteoglycan (PG) versican. In the versican-null lung, there are no such matrices, and numbers of infiltrated leukocytes do not increase (12). The HA–versican interaction is important for the recruitment of inflammatory cells including neutrophils, macrophages, and T cells (13, 14). An early work showed a significant increase in CS synthesis in the normal lung after intravenous administration of a single dose of endotoxin (15).

Therefore, PGs accumulate in inflammatory areas and induce inflammatory cell infiltration. Increasing evidence suggests an anti-inflammatory activity of CS through suppression of pro-inflammatory cytokine activities (16–18). While the structure–function relationship of CS is controversial, we aim to introduce the latest information on the role of CS in inflammation.

## Structure of Chondroitin Sulfate

**Figure 1:**
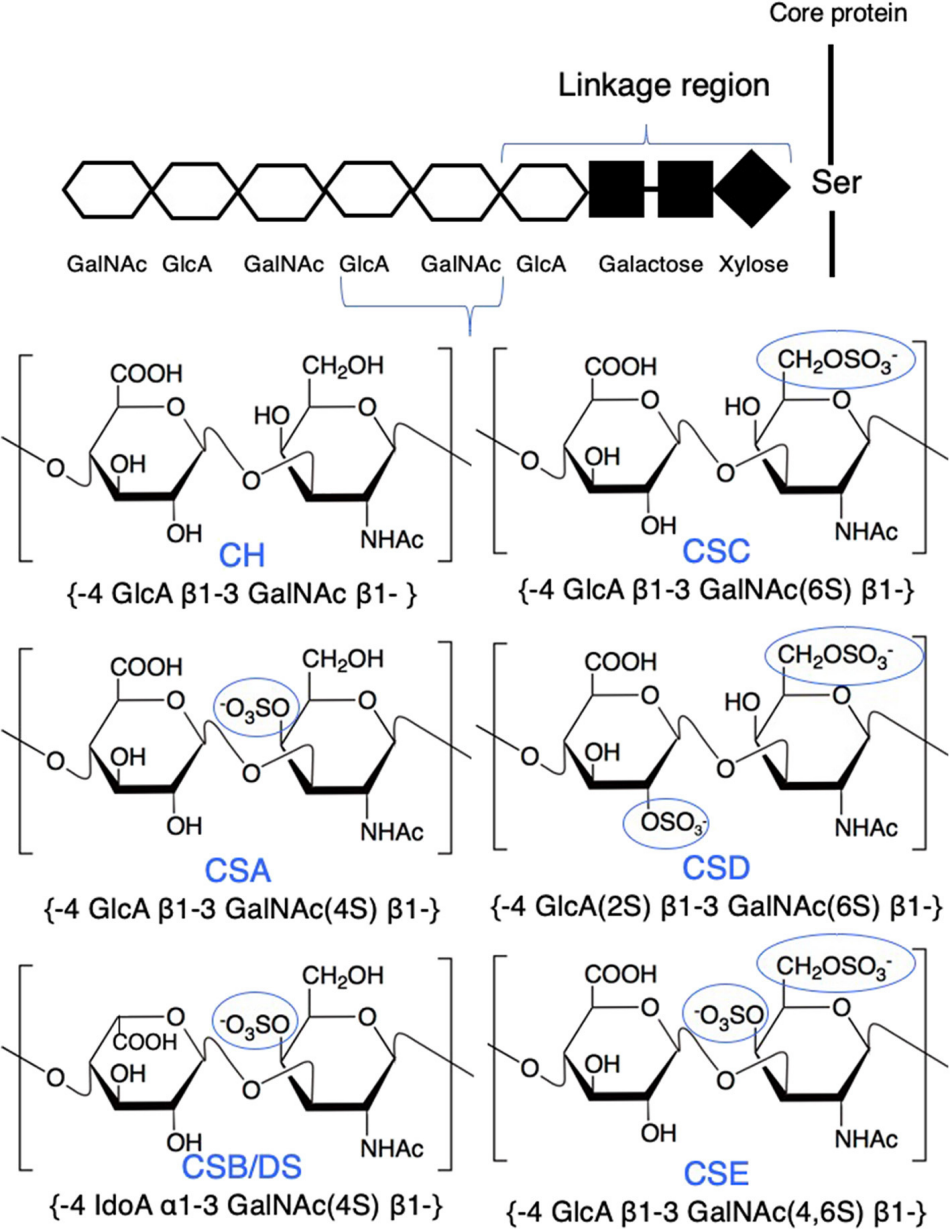
Structure of major chondroitin sulfate (CS) disaccharides. CS is linked to a serine residue of a core protein *via* a linkage region. Repeating disaccharides form a linear polysaccharide chain, which is modified with sulfate groups at varying positions on sugar residues. The major disaccharide structures of CS are as follows: a non-sulfated unit (CH, GlcA-GalNAc); a monosulfated unit at the C-4 position of the GalNAc residue (CSA, GlcA-GalNAc4S); a monosulfated unit at the C-6 position of the GalNAc residue (CSC, GlcA-GalNAc6S); a disulfated unit at the C-2 position of GlcA and the C-6 position of the GalNAc residue (CSD, GlcA2S-GalNAc6S); and a disulfated unit at the C-4 and the C-6 positions of the GalNAc residue (CSE, GlcA-GalNAc4S6S). Certain GlcA residues are epimerized to IdoA (CSB or DS, IdoA-GalNAc4S).

Chondroitin sulfate (CS) is a natural biomacromolecule abundantly distributed in virtually all invertebrates and vertebrates and involved in many biological processes (19, 20). Based on its structure, chain length, and sulfation patterns, CS provides specific biological functions at molecular, cellular, and organ levels, such as cell adhesion, cell division and differentiation, morphogenesis, organogenesis, and neural network formation (6, 21). CS is composed of a repeating glucuronic acid (GlcA) and *N*-acetylgalactosamine (GalNAc) and modified with sulfate groups at varying positions on sugar residues. The major disaccharide structures of CS are as follows: a non-sulfated unit (CH, GlcA-GalNAc), a monosulfated unit at the C-4 position of the GalNAc residue (chondroitin 4-sulfate: CSA, GlcA-GalNAc4S), a monosulfated unit at the C-6 position of GalNAc (chondroitin 6-sulfate: CSC, GlcA-GalNAc6S), a disulfated unit at the C-4 and C-6 positions of GalNAc (chondroitin 4, 6-sulfate: CSE, GlcA-GalNAc4S6S), a disulfated unit at the C-2 position of GlcA and the C-4 position of GalNAc (chondroitin 2,4-sulfate, GlcA2S-GalNAc4S), a disulfated unit at the C-2 position of GlcA and the C-6 position of GalNAc (chondroitin 2, 6-sulfate: CSD, GlcA2S-GalNAc6S), and a trisulfated unit at the C-2 position of GlcA and the C-4 and C-6 positions of GalNAc (GlcA2S-GalNAc4S6S). Certain GlcA residues are epimerized to iduronic acid (IdoA); the chain containing IdoA residues is designated as CSB or DS (Figure 1).

Thus, CS possesses a heterogeneous structure and physical–chemical profile in different species and tissues and is responsible for the various and more specialized functions in the extracellular matrix (ECM). To understand the structure–function relationship of CS, our group developed a sequence determination method of synthesized CS dodecasaccharides (22) and generated a CS library *via* chemo-enzymatic synthesis (23). This CS library showed that CSA interacts with a malarial variant surface antigen 2-CSA (VAR2CSA) protein and may potentially serve as a target in therapeutic strategies against placental malaria (24).

## The Effect of Chondroitin Sulfate on Antigen-Presenting Cells (Idem for Macrophages and Dendritic Cells)

**Figure 2:**
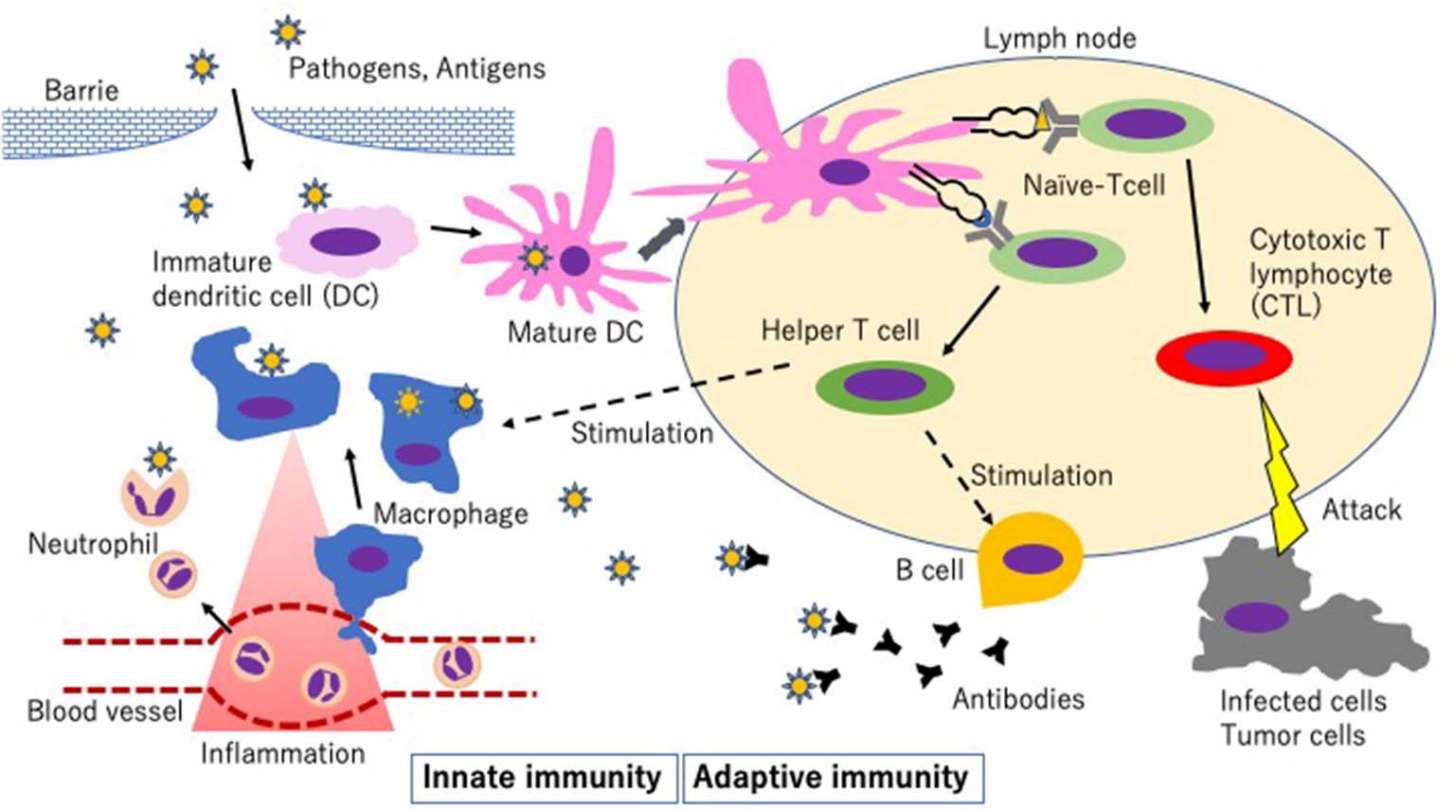
Schematic diagram of innate immunity and adaptive immunity. Step 1: Activation of pattern recognition receptors (PRRs) on tissue-resident macrophages and/or dendritic cells (DCs) by pathogen-associated molecular patterns (PAMPs) resulting in activation of the innate immune response and the following events. 1. Migration of monocytes that mature into recruited macrophages and neutrophils in the systemic circulation. 2. Maturation of DCs, which then migrate to the lymph node. Step 2: Processing of the antigens and presentation of an antigen on major histocompatibility complex I (MHC-I) or MHC-II to the T cell receptor on T cells. Step 3: Development of adaptive immunity.

Antigen-presenting cells (APCs), including macrophages, dendritic cells (DCs), and B cells, trigger innate immunity by different mechanisms (Figure 2). In the innate immune system, the recognition of extracellular pathogen is mainly mediated by macrophages and DCs in the mononuclear phagocyte system. They recognize pathogen-associated molecular patterns (PAMPs) brought by microbes and damage-associated molecular patterns (DAMPs) produced by damaged host cells through antigen-specific surface receptors, including pattern recognition receptors (PRRs) (25). Toll-like receptors (TLRs) represent a major PRR family. Once their extracellular domains bind PAMPs or DAMPs, the TLRs trigger an intracellular signaling pathway to activate various transcription factors such as nuclear factor-κB (NF-κB). After recognizing their specific molecular patterns, APCs internalize antigens by phagocytosis, process them, and display the fragment of antigen on their surface with major histocompatibility complex (MHC) (26, 27). In general, macrophages remain at the inflammatory sites to eliminate pathogens and apoptotic cells by phagocytosis and clearance and produce pro-inflammatory cytokines. In contrast, DCs can travel to the draining lymph nodes and stimulate T cells (28, 29).

### The Role of Chondroitin Sulfate in Macrophages

Macrophages are typically activated in a pro-inflammatory phenotype (M1) or an anti-inflammatory phenotype (M2). Furthermore, M2 macrophage is classified into four subdivisions, M2a, M2b, M2c, and M2d, depending on the applied stimuli and their protein expression profile (30). M1 secretes various pro-inflammatory cytokines and chemokines, such as tumor necrosis factor-alpha (TNF-α), interleukin (IL)-1β, IL-6, IL-8, etc., to scavenge pathogens (31), and M2 produces inflammation inhibitory factors, such as IL-10 and Arginase 1, to avoid excessive inflammation and promote tissue repair (32). Most tissue-resident macrophages are not originated from circulating hematopoietic stem cell-derived monocytes but developed from embryonic precursors including the yolk-sac macrophage or fetal liver monocytes (33, 34).

The anti-inflammatory activity of CS has been studied concerning macrophages. CS influences inflammatory processes by limiting NF-κB signaling (16, 35) and also inhibits IL-1β-induced liberation of pro-inflammatory genes, such as IL-6, nitric oxide synthase 2, and prostaglandin E2 synthase (36, 37). Further, CS blocks lipopolysaccharide (LPS) binding to CD44 on rat bone marrow-derived macrophages to inhibit the LPS/CD44/NF-κB pathway (38). In CS structure, CSA and its *N*-deacetylated derivative can activate NF-κB in macrophages and induce TNF-α production (39). In contrast, CSC attenuates the inflammatory response in macrophages *via* suppression of NF-κB nuclear translocation (40). Moreover, CSA or CSC inhibited the LPS-induced expression of TNF-α, IL-1β, IL-6, and nitric oxide (NO) on bone marrow-derived macrophages (36). The functions of the CS sulfated structures on macrophages are still in argument.

### The Role of Chondroitin Sulfate in Dendritic Cells

Dendritic cells (DCs) are more potent APCs than macrophages and the major instructors of T cells (41). In local tissues, including skin and intestine, DCs recognize PAMPs or DAMPs through a large variety of PRRs and phagocytose the antigens and become activated during this process. A variety of factors, such as IL-1 (42) and TNF-α (43), whole bacteria and microbial cell wall component LPS (44), CpG motifs in bacterial DNA (45), haptens (44), and apoptotic cells (44), stimulate DC maturation and promote the expression of MHC-antigen-presenting and costimulatory molecules. The mature DCs migrate to nearby lymphoid organs to present the peptide to naive T cells in a complex with MHC proteins (28). Naive T cells can differentiate into several types of effector T cells *via* MHC class II complex and CD8^+^ T cells *via* MHC class I complex (41, 46–48). Effector cells include four types of helper T cells, namely, Th1, Th2, T_FH_, Th17, and regulatory T cells, depending on the cytokines they encounter (49, 50). The mature DCs are divided into three major subsets of conventional DCs (cDC1s and cDC2s) and plasmacytoid DCs (pDCs). cDC1 has a high intrinsic capacity to cross-present antigens *via* MHC class I to activate CD8^+^ T cells and to promote Th1. cDC2 influences a wide range of naive T cell differentiation to Th1, Th2, Th17, and CD8^+^ T cells (51). Although pDCs are specialized to respond to viral infection with a massive production of type I interferons (IFNs), they also act as APCs and control T cell responses (28).

Both sulfate group content and position in CS are important for Th1 cell-promoted activity of murine splenocytes in terms of cytokine production (52). CSA exhibits the highest cytokine production activity in murine splenocytes. In contrast, CSE decreases Th1-promoted and Th2-inhibitory activity (53). A CSPG fraction mainly of aggrecan extracted from salmon nasal cartilage attenuates the severity of experimental autoimmune encephalomyelitis (EAE), by suppressing the differentiation of the Th17 lineage, and enhances regulatory T cell expansion (54). In EAE mice, treatment with CSD disaccharides inhibits the expression of IFN-γ in the brain (55). CSD treatment also obviously alleviates the clinical symptoms of EAE by limiting T cell infiltration and microglial activation. However, CSA treatment exacerbates EAE symptoms by stimulating T cell infiltration in the central nervous system (CNS) and inducing their differentiation into Th1 and Th17 lineages (56). Moreover, CSC displayed a neuroprotective effect in EAE and may inhibit the spread of pathogenic T cells in the CNS (57). DCs play a pivotal role in promoting unbalanced active immune responses, resulting in the progression of autoimmune diseases. These results suggest that CS could regulate DC function, which leads to designated T cell differentiation.

## Receptors of Chondroitin Sulfate on Antigen-Presenting Cells

### Toll-Like Receptors

The components of ECM are recognized by TLRs as DAMPs. To date, 13 mammalian TLRs have been identified. Each TLR recognizes specific PAMPs and DAMPs including lipopeptides for TLR1, TLR2, and TLR6, LPS for TLR4, bacterial flagellin for TLR5, dsRNA for TLR3, ssRNA for TLR7 and TLR8, and DNA for TLR9 (58). TLR2 and TLR4 can also be activated by endogenous ligands or DAMPs (59, 60). A small proteoglycan biglycan stimulates macrophage activation *via* TLR2 or TLR4. The effect is significantly reduced in TLR4-mutant and TLR2^−/−^ macrophages and abolished in TLR2^−/−^/TLR4-mutant macrophages (61). In cancer-associated inflammation, tumor-derived versican causes a dysfunction of DCs *via* activation of their TLR2 (62). Versican also facilitates Lewis lung carcinoma metastasis through TLR2 and its co-receptor TLR6 (63). Although versican and biglycan are CSPGs, these reports did not mention whether CS or core protein is responsible for these effects. In macrophage-like cell line, smaller sized CSA or its disaccharides suppress IL-6 secretion, whereas no such size-dependent suppression was apparent for CSC (21). CSA and CSC significantly inhibit NF-κB activity and inflammatory cytokines *via* TLR4 (64). To elucidate the structure–function relationship between CSs and TLRs, further studies are expected.

### Receptor-Type Protein Tyrosine Phosphatase Sigma

The receptor-type protein tyrosine phosphatase sigma (RPTPσ) is found as an inhibitor of axonal growth and nerve regeneration with CS (65), and is a cell-surface protein that has intracellular tyrosine phosphatase activity and extracellular domains. RPTPσ is one of the type IIa RPTPs, and others are leukocyte common antigen-related (LAR) and RPTPδ. Out of the three, RPTPσ is expressed in several immune cells, including DCs, and is essential for regulating immune cell activation, cytokine production, and inflammation (66, 67). RPTPσ interacts with both CS and HP/HS in the nervous system, with a resembling binding affinity (65, 68, 69). CS and HP/HS compete for the same binding site of RPTPσ in the first Ig-like domain and result in opposing effects on axon elongation. Crystallographic analysis suggests that CS can prevent RPTPσ dimerization, while HS induces RPTPσ clustering (70). Using a biotin-conjugated CS GAG library composed of chemoenzymatically synthesized CS species, RPTPσ binds to CSE with 10-kDa molecular mass, but not to CSA or CSC (71).

RPTPσ acts as a receptor to inhibit autoimmune-related inflammation by preventing DC hyperactivation (66). Since RPTPσ is crucial for suppressing immune responses mediated by DCs, the CS function through RPTPσ might contribute to various immune-related diseases.

### CD44

CD44 is a transmembrane glycoprotein that exhibits extensive molecular heterogeneity. The CD44 ectodomain is responsible for binding HA; low-molecular-weight HA triggers TLR-mediated inflammation (72–74). In macrophages, biglycan and HA induce autophagy through interaction with CD44 (75, 76). Besides biglycan and HA, versican (77), osteopontin (78, 79), and macrophage migration inhibitory factor (80) are also ligands of CD44. The cytoplasmic domain modulates inflammatory signaling in a ligand-dependent manner *via* TLR2 and TLR4 activity (76, 79, 80).

CD44 is dramatically overexpressed on the surface of activated macrophages found at sites of inflammation, as such, it has been widely used as a receptor for targeted drug delivery (81–83). Given the relationship between CS and CD44, CS can be used to modify nanoparticles to enhance the cellular uptake of nanoparticles *via* CD44-mediated endocytosis (84, 85). Remarkably, CS exhibits a high affinity for CD44 and facilitates cell internalization *via* CD44-mediated endocytosis (86, 87).

## The Role of Core Protein in Chondroitin Sulfate Proteoglycan

### Versican

Versican is a large CSPG in the ECM and comprises a core protein of approximately 400 kDa, with approximately 20 attachment sites for CS side chains (88, 89). The core protein contains an N-terminal G1 domain and a C terminal G3 domain, with CS-attached domains between the two globular domains, and comprises four alternative splicing forms (V0, V1, V2, and V3). Versican interacts with HA at the G1 domain and other ECM molecules at the G3 domain. Versican acts on inflammatory responses as a DAMP *via* cell surface proteins such as CD44 (77, 90), CD162 (91), TLR2, TLR6, and CD14 (63, 92).

The versican gene is upregulated in monocytes/macrophages in some pro-inflammatory states, such as myocardial infarction (93), coronary stenosis (94), and autoimmunity (95–97). Experiments performed *in vitro* using classically activated murine bone marrow-derived macrophages treated with LPS showed that M1 type of macrophages exhibited a high expression level of versican mRNA, as well as versican accumulation (98). In human monocyte-to-macrophage differentiation and polarization, the versican gene expression level of M1 macrophages is higher than that of M2 macrophages (99). CSA and V1 core protein were upregulated in the perivascular cuff of multiple sclerosis and EAE and migration of leukocytes including macrophages across the glia limitans into the CNS parenchyma (100).

Versican secreted by Lewis lung carcinoma cells interacts with TLR2 on DCs and sensitizes DCs to respond with IL-6 and IL-10 by increasing the expression of cell surface receptors for IL-6 and IL-10 (62). This result indicates the protumor properties of intact versican. On the other hand, versikine, a degradation product of versican, also interacts with TLR2 on macrophages and acts with antitumor properties (101–103). Versikine is a 70-kDa N-terminal fragment (104), including the G1 domain and lacking CS-attached and the G3 domains. As the intact versican possesses a lot of functional domains including CS, there might be different manners to interact with TLR2.

We previously showed that embryonic fibroblasts, which express the mutant versican lacking the A-subdomain of the G1 domain, attain cell senescence (105). As there is a higher content of CSC and CSE in their conditioned media, the CS composition of the mutant versican could be altered (106). Changes in CS composition are probably caused by the changed core proteins.

### Biglycan

The small leucine-rich proteoglycans (SLRPs) in the ECM regulate cell function in the inflammatory sites. Their core proteins have leucine-rich repeat (LRR) motifs and are attached with the CS and/or DS side chains. Biglycan is one of the SLRPs (107, 108) and consists of a 42-kDa core protein containing 10 LRRs and up to two covalently bound CS and/or DS side chains. Biglycan interacts with types I, II, III, and VI collagen and regulates collagen fibrillogenesis (109–112). This regulation is mediated by the core protein, whereas the CS/DS side chains maintain interfibrillar space by extending outward from the protein core (113). The soluble form of biglycan initiates and perpetuates the inflammatory response by activating TLR2 and TLR4, and biglycan-deficient mice are less susceptible to death caused by TLR2- or TLR4-dependent sepsis (61). It is important that the biglycan core protein directly binds to CD44, and the GAG side chains enhance this interaction (75). Both the biglycan core protein and GAG side chain are also necessary for IL-1β maturation of macrophages (114). Sulfated CS/DS side chains are also implicated in lipid retention by direct interaction with low-density lipoprotein (LDL). In atherosclerotic plaque, LDL colocalizes with biglycan. The interaction between LDL and PGs promotes modification and aggregation of LDL, and uptake of LDL by macrophages leads to foam cell formation (115). Therefore, biglycan core protein plays a pivotal role in the suppression of inflammation and the transition from innate to adaptive immunity (116). Recently, an excellent review reported that biglycan, HA, and versican as the matrix-derived DAMPs regulate TLR-, CD14-, and CD44-signaling cross talk between inflammation and autophagy (85).

### Syndecan

The syndecan (SDC) family of cell surface heparan sulfate proteoglycan mediates cell–cell and cell–matrix interactions *via* the GAG chains and is also important in the regulation of inflammation. SDCs are type I transmembrane proteoglycans and consist of four distinct members whose ectodomain varies among the members. SDC1 is widely expressed in epithelia and leukocytes. SDC2 is mainly expressed in endothelial cells and fibroblasts. SDC3 is expressed in neural tissues (117). SDC4 is abundant in many cell types and a soluble protein isoform lacking the transmembrane and cytoplasmic domains (118). Though HS is usually covalently attached to SDCs, SDC1 and SDC3 also bear two CS chains (119). Deletion of SDC1 leads to an exacerbation of allergic asthma (120). SDC4-deficient mice exhibit increased susceptibility to endotoxin shock (121). Although ubiquitous SDC4 expression is low in a steady state, SDC4 expression elevates in mice post-LPS stimulation of macrophages (122). Immature DCs express increased glypican-1 and SDC1 compared to mature DCs, whereas mature DCs express glypican-3, which was not present in immature DCs (123). Research has illustrated that SDC1 on DC negatively regulates DC migration; therefore, lower SDC1 expression levels are often associated with mature DCs (124). Furthermore, the functional switch from SDC1 to SDC4 expression during DC maturation controls DC motility and subsequent migration from peripheral sites to lymphoid tissues (125).

## Conclusion

This mini-review described regulation of CS on APCs with their different structures through their specific receptors at inflammatory sites. CS exhibits both pro- and anti-inflammatory activities with their heterogeneous structures. Even with the same structure, CS affects differently depending on the target cells and their microenvironments. Regarding their sulfation patterns, CSC and CSD have anti-inflammatory activity, whereas CSA has both pro- and anti-inflammatory activities. In many cases, CSE has a potential anti-inflammatory activity, although a recent report suggests its stimulatory effect on tumor progression with the pro-inflammatory activity (6). Regarding the CS chain length, short chains such as oligosaccharides or disaccharides mostly activate inflammation, whereas the long chains serve as anti-inflammatory factors. CS structure and length may vary by the different core proteins and their expressing cells. To understand detailed CS functions in immunity, further investigations into the structure–function relationship of CS are needed.

## Author Contributions

SH and HW contributed to the conception and design of the study. SH wrote the first draft of the manuscript and wrote sections of the manuscript. All authors contributed to manuscript revision, read and approved the submitted version.

SH took primary responsibility for communication with the journal and editorial office during the submission process, throughout peer review and during publication, ensuring that the submission adheres to all journal requirements including, but not exclusive to, details of authorship, study ethics and ethics approval, clinical trial registration documents and conflict of interest declaration, and available post-publication to respond to any queries or critiques.

### Conflict of Interest

The authors declare that the research was conducted in the absence of any commercial or financial relationships that could be construed as a potential conflict of interest.

## Abbreviations

CS: chondroitin sulfate
GAG: glycosaminoglycan
HA: hyaluronan
PG: proteoglycan
CSPG: chondroitin sulfate proteoglycan
HS: heparan sulfate
DS: dermatan sulfate
HP: heparin
KS: keratan sulfate
GlcA: glucuronic acid
GalNAc: *N*-acetylgalactosamine
CSA: chondroitin 4-sulfate
CSC: chondroitin 6-sulfate
CSD: chondroitin 2,6-sulfate
CSE: chondroitin 4,6-sulfate
IdoA: iduronic acid
ECM: extracellular matrix
VAR2CSA: variant surface antigen 2-CSA
APC: antigen-presenting cell
DC: dendritic cell
PAMP: pathogen-associated molecular pattern
DAMP: damage-associated molecular pattern
PRR: pattern recognition receptor
TLR: Toll-like receptor
MHC: major histocompatibility complex
TNF-α: tumor necrosis factor-alpha
IL: interleukin
LPS: lipopolysaccharide
IFN: interferon
EAE: experimental autoimmune encephalomyelitis
CNS: central nervous system
RPTPσ: receptor type of protein tyrosine phosphatase sigma
LAR: leukocyte common antigen-related
SLRP: small leucine-rich proteoglycan
LRR: leucine-rich repeat
LDL: low-density lipoprotein
SDC: syndecan.
